# Systemic alterations play a dominant role in epigenetic predisposition to breast cancer in offspring of obese fathers and is transmitted to a second generation

**DOI:** 10.1038/s41598-021-86548-w

**Published:** 2021-04-01

**Authors:** Camile C. Fontelles, Raquel Santana da Cruz, Alexandra K. Gonsiewski, Ersilia Barin, Volkan Tekmen, Lu Jin, M. Idalia Cruz, Olivier Loudig, Anni Warri, Sonia de Assis

**Affiliations:** 1 Department of Oncology, Lombardi Comprehensive Cancer Center, Georgetown University, 3970 Reservoir Road, NW, The Research Building, Room E410, Washington, DC 20057 USA; 2 Center for Discovery and Innovation, Hackensack Meridian Health (HMH), Nutley, NJ USA; 3 Institute of Biomedicine, University of Turku Medical Faculty, 20014 Turku, Finland

**Keywords:** Cancer, Developmental biology, Oncology

## Abstract

We previously showed that environmentally-induced epigenetic inheritance of cancer occurs in rodent models. For instance, we reported that paternal consumption of an obesity-inducing diet (OID) increased breast cancer susceptibility in the offspring (F1). Nevertheless, it is still unclear whether programming of breast cancer in daughters is due to systemic alterations or mammary epithelium-specific factors and whether the breast cancer predisposition in F1 progeny can be transmitted to subsequent generations. In this study, we show that mammary glands from F1 control (CO) female offspring exhibit enhanced growth when transplanted into OID females compared to CO mammary glands transplanted into CO females. Similarly, carcinogen-induced mammary tumors from F1 CO female offspring transplanted into OID females has a higher proliferation/apoptosis rate. Further, we show that granddaughters (F2) from the OID grand-paternal germline have accelerated tumor growth compared to CO granddaughters. This between-generation transmission of cancer predisposition is associated with changes in sperm tRNA fragments in OID males. Our findings indicate that systemic and mammary stromal alterations are significant contributors to programming of mammary development and likely cancer predisposition in OID daughters. Our data also show that breast cancer predisposition is transmitted to subsequent generations and may explain some familial cancers, if confirmed in humans.

## Introduction

Genetic predisposition explains most but not all familial diseases, including breast cancer^1^. It is increasingly evident that epigenetic inheritance of disease can also occur and may explain some inherited conditions. There is strong indication that, at conception, parents pass more than genetic material to their offspring. They also transmit a molecular memory of past environmental exposures^2,3^ which can result in offspring’s predisposition for certain chronic diseases^4^.

Life-style and environmental insults have been shown to reprogram the sperm epigenome in humans and in animal models^5,6^. Recently published studies demonstrated that the small RNA load in paternal sperm can convey phenotypes to the progeny^3,7–9^. Some of those reports implicate t-RNA fragments (tRFs)—which are the most abundant small RNA sub-type in sperm—in the transmission of environmentally-induced information from fathers to offspring and show that they can recapitulate disease phenotypes^7–10^.

Because mammary gland development starts during the fetal stage, multiple studies report that maternal exposure during gestation can epigenetically reprogram the daughters’ mammary tissue and increase breast cancer development^11–14^. However, a role for paternal exposures in modulating breast cancer predisposition in offspring has emerged in recent years. We recently showed that paternal obesity, malnutrition and consumption of a high-fat diet all lead to increased breast cancer development in offspring^15–17^, a phenotype associated with changes in normal mammary gland development. We also found that a recurrent phenotype accompanying offspring’s cancer predisposition is metabolic dysfunction^16–18^, raising the possibility that paternally-induced cancer development could be a function of both systemic effects as well as tissue specific changes.

Paternal effects on the F1 generation include alterations in the germline epigenome^19^, suggesting that disease traits in offspring could be passed on to future generations. Indeed, it has been reported that paternally-induced phenotypes observed in the F1 can be transmitted to the F2 generation^19,20^. There is also evidence for environmentally-induced intergenerational programming of disease from human cohorts: A recent study reported an association between paternal nutritional status and increased cancer mortality in subsequent generations^21^. It is not clear, however, whether paternally-induced breast cancer predisposition observed in daughters can be transferred to successive generations.

Here, we used a mouse model of paternal obesity and aimed to address the distinct contributions of systemic effects and local tissue-confined factors on mammary gland and breast cancer development in daughters of obese fathers. We also investigated whether the breast cancer predisposition observed in daughters of obese fathers^15,16^ could be transmitted to granddaughters.

## Results

### Offspring of OID fathers have impaired metabolic function and altered mammary gland development

**Figure 1:**
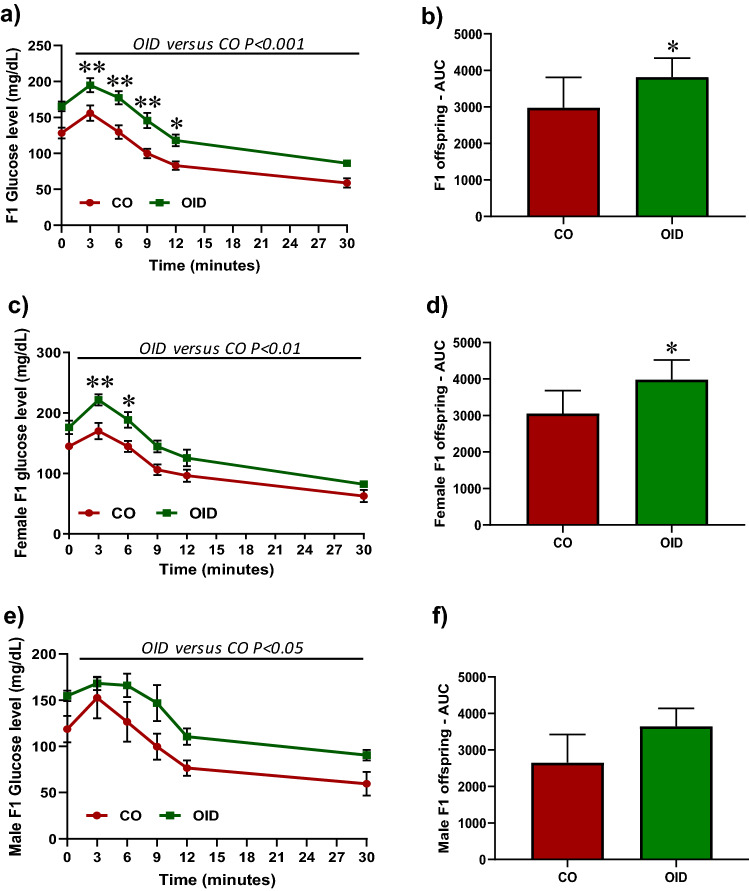
Paternal OID causes metabolic disturbance in offspring. Insulin tolerance test (ITT) and area under curve (AUC) in all gender (**a**,**b**), female (**c**,**d**) and male (**e**,**f**) F1 offspring (n = 7–8/gender/group) from CO and OID-fed fathers. The data are expressed as mean ± SEM. Significant differences versus the control group were determined by two-way ANOVA followed by post-hoc analysis. *P ≤ 0.05; **P ≤ 0.01.

We previously reported that paternal consumption of obesity-inducing diets (OID) at the pre-conception window increased female offspring’s susceptibility to breast cancer^15,16^. In those studies, we also described mammary gland morphological changes as well as metabolic dysfunction—a phenotype also reported by others—in offspring of obese fathers^16,18,19,22^. Our present results corroborate our previous findings as OID offspring (F1) displayed impaired metabolic function with both F1 males and females showing significantly higher glucose levels in an insulin tolerance test (ITT) compared to CO offspring (*P* = 0.002, *P* = 0.011, Fig. 1a–f). In addition, mammary glands of OID daughters also showed increased number of terminal end buds (TEB), higher epithelial branching and elongation, although only the last parameter reached statistical significance compared to CO (Table S1). Those phenotypes were not associated with body weight gain (Fig. S1) as OID offspring weights either did not differ from or were lower than those of CO offspring.

### Systemic effects play a larger role in normal mammary tissue and mammary tumor growth in offspring of OID fathers

**Figure 2:**
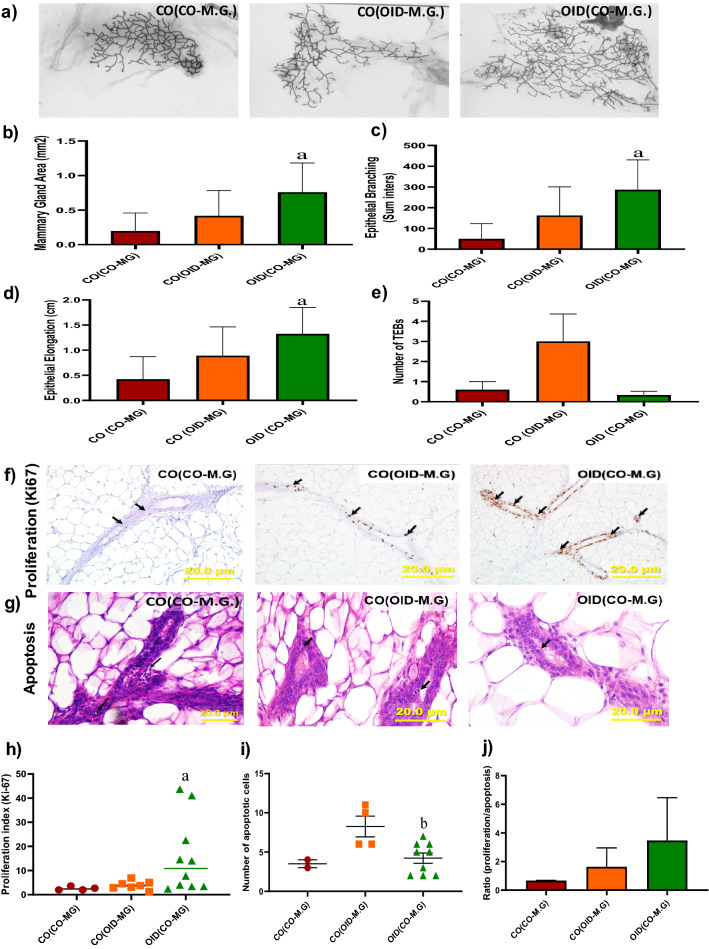
Development of transplanted mammary glands in CO or OID daughters (F1). Histological depiction of transplanted mammary gland in (**a**) [CO(CO-M.G)], [CO(OID-M.G)], and [OID(CO-M.G)] groups. Graphs below show values for mammary gland area (**b**), epithelial branching (**c)**, epithelial elongation (**d**) and number of terminal end buds (TEB) (**e**), (**b**–**e**, n = 6–13); Photomicrograph of Ki-67 immunostaining (**f**) (20×, staining indicated by arrows) and apoptotic cells (**g**) (H&E morphological assessment, 40×, cells indicated by arrows). Graphs below show proliferation index (**h**), number of apoptotic cells (**i**) and proliferation/apoptosis ratio (**j**), (**f**–**i**, n = 4–12). The data are expressed as mean ± SEM. Significance differences between groups were determined by one-way ANOVA followed by post-hoc analysis (mammary gland area, branching density, epithelial elongation, number of TEBs, cell proliferation and apoptosis numbers). “a” indicates statistically significant difference (*P* ≤ 0.05) between OID(CO-M.G) and CO(CO-M.G); “b” indicates statistically significant difference (*P* ≤ 0.05) between OID(CO-M.G) and CO(OID-M.G).

Next, we examined the contributions of systemic alterations and mammary tissue specific factors (stroma vs. epithelium) to the increased breast cancer development in offspring of obese fathers. In the first experiment, female offspring of either CO or OID-fed males underwent a mammary gland transplantation surgery. CO mammary glands transplanted into OID females [OID(CO.MG)] exhibited accelerated development (Fig. 2a–e) as shown by higher mammary gland area (*P* = 0.032, Fig. 2b), higher mammary branching and higher epithelial elongation (*P* = 0.014; *P* = 0.008, respectively, Fig. 2c,d), but not higher number of TEBs (Fig. 2e), compared to CO females that received a CO mammary gland [CO(CO.MG)]. This phenotype was associated with a higher proliferation index and lower apoptotic rates compared to [CO(CO.MG)]) and [CO(OID.MG)] (*P* = 0.021 and *P* = 0.026, respectively; Fig. 2f–j). While OID mammary glands transplanted into CO females [CO(OID.MG)] showed slightly higher mammary gland area, mammary branching and epithelial elongation and number of TEBS (Fig. 2b–e) compared to [CO(CO.MG)], results did not reach statistical significance.

**Figure 3:**
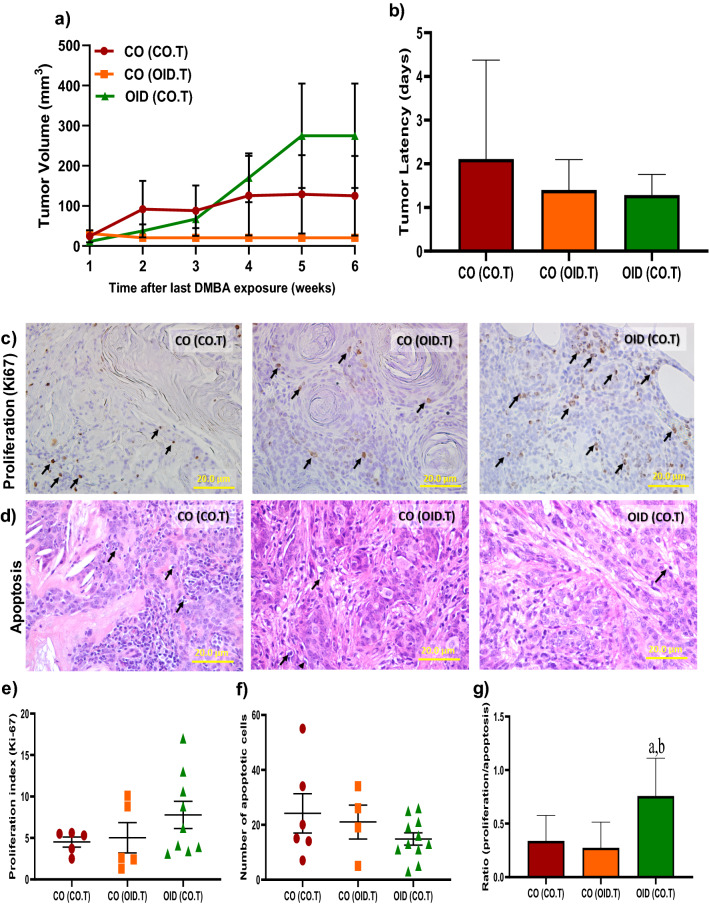
Development of transplanted mammary tumors in CO or OID daughters (F1). Tumor volume (**a**) and latency (**b**) (**a**,**b**, n = 10–18/group) in [CO(CO-M.G)], [CO(OID-M.G)], and [OID(CO-M.G)] groups after a 6-week monitoring period. Photomicrograph of Ki-67 immunostaining (**c**) (20×, staining indicated by arrows) and apoptotic cells (**d**) (H&E morphological assessment, 20×, cells indicated by arrows). Graphs below show proliferation index (**e**), number of apoptotic cells (**f**), and proliferation/apoptosis ratio (**g**), (**c**–**g**, n = 3–11/group). The data are expressed as mean ± SEM. Significance differences between groups were analyzed by repeated measures ANOVA (mammary tumor volume) and one-way ANOVA (tumor latency, proliferation index and number of apoptotic cells) followed by post-hoc analysis. “a” indicates statistically significant difference (*P* ≤ 0.05) between OID(CO.T) and CO(CO.T); “b” indicates statistically significant difference (*P* ≤ 0.05) between OID(CO.T) and CO(OID.T).

Given that both the mammary microenvironment and systemic response could play a role in tumor progression, we also asked whether the metabolic-induced mammary stroma milieu could affect the growth potential of tumors. Thus, in our second experiment, a DMBA-induced mammary tumor of F1 female offspring from CO (donor) was transplanted into the fat pad of a CO or OID female offspring (host) and vice versa. Tumor growth was followed for 6–8 weeks post-surgery. While not statistically significant due to large intra-group variation, CO tumors transplanted into OID females [OID(CO.T)] seemed to have improved growth (Fig. 3a,b) compared to CO or OID tumors transplanted in CO females [CO(CO.T) and CO (OID.T)]. In line with that, [OID(CO.T)] tumor also showed significantly increased cell proliferation to apoptosis ratio, compared to both [CO(CO.T)] and [CO (OID.T)] (*P* = 0.043, *P* = 0.032, respectively; Fig. 3c–g).

### Consumption of OID alters the tRF content in sperm of fathers (F0) and their sons (F1)

**Figure 4:**
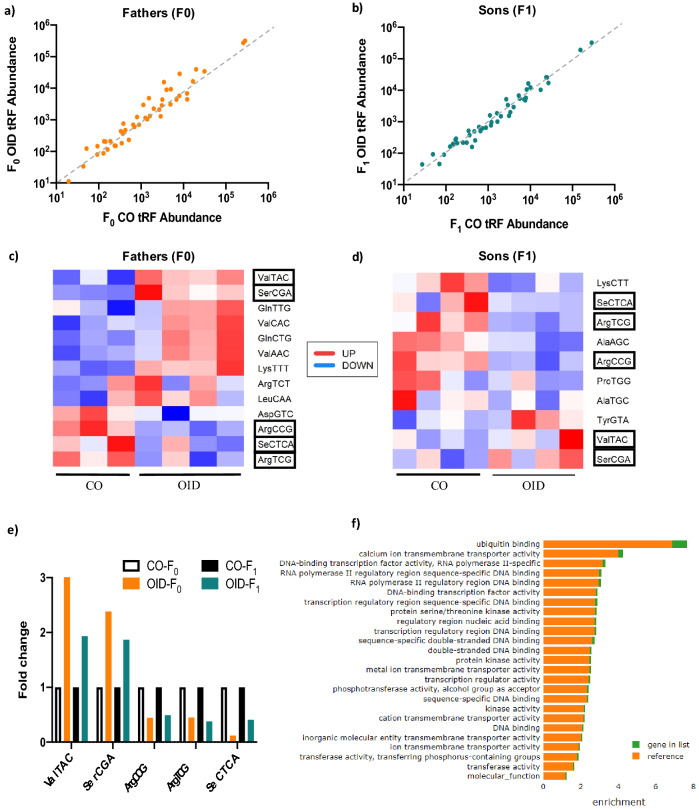
Paternal OID reprograms the sperm small non-coding RNA load in fathers (F0) and sons (F1). (**a**,**b**) Scatterplot of sperm tRNA fragments (tRF) from OID (y-axis) fathers (F0, **a**) and OID sons (F1, **b**) versus their respective controls (CO, x-axis) (n = 3–4/group) assessed by RNA-seq. (**c**,**d**) Heat-map showing differentially expressed tRNA fragments (tRFs) in sperm from OID fathers (**c**) and sons **(d**) compared to CO, highlighting overlapping tRFs in F0 and F1 (boxes). (**e**) Levels (fold change) of the 5 tRFs with overlapping differential expression in both OID fathers(F0) and sons (F1) compared to CO. (**f**) Gene ontology molecular functions significantly enriched in the targets of ValTAC, SerCGA, ArgCCG, ArgTCG and SeCTCA. Heatmaps were created using gplots (v3.1.1) package: https://cran.r-project.org/web/packages/gplots/index.html.

Recent studies have suggested that sperm non-coding RNAs play a role in transmitting environmentally-induced information from fathers to offspring. Transfer RNA fragments or tRFs make up the majority of small RNAs in mature sperm and can recapitulate the effects of paternal obesity in offspring^3^. As reported before, GlyGCC and GlutCTC were the most abundant tRFs in sperm of both fathers (F0) and their male offspring (F1), representing about 70% of all tRFs (Fig. 4a,b)^8,19^. We also found that consumption of OID altered specific tRFs in both father (Fig. 4c) and sons (Fig. 4d), with five tRFs overlapping between the two generations (Fig. 4e, Fig. S2): Levels of ValTAC and SerCGA were increased while those of ArgCCG, ArgTCG and SeCTCA were decreased in sperm of OID F0 and F1 males compared to CO. Because tRFs have been shown to play a role in the regulation of gene expression^3,6^, we then evaluated possible targets of these five tRFs. Putative targets of these five tRFs were significantly enriched for molecular functions related to DNA binding, transcription factor activity, transcriptional regulation, and transmembrane transporters among others (Fig. 4f).

### Breast cancer predisposition in OID daughters is transmitted to a second generation

**Figure 5:**
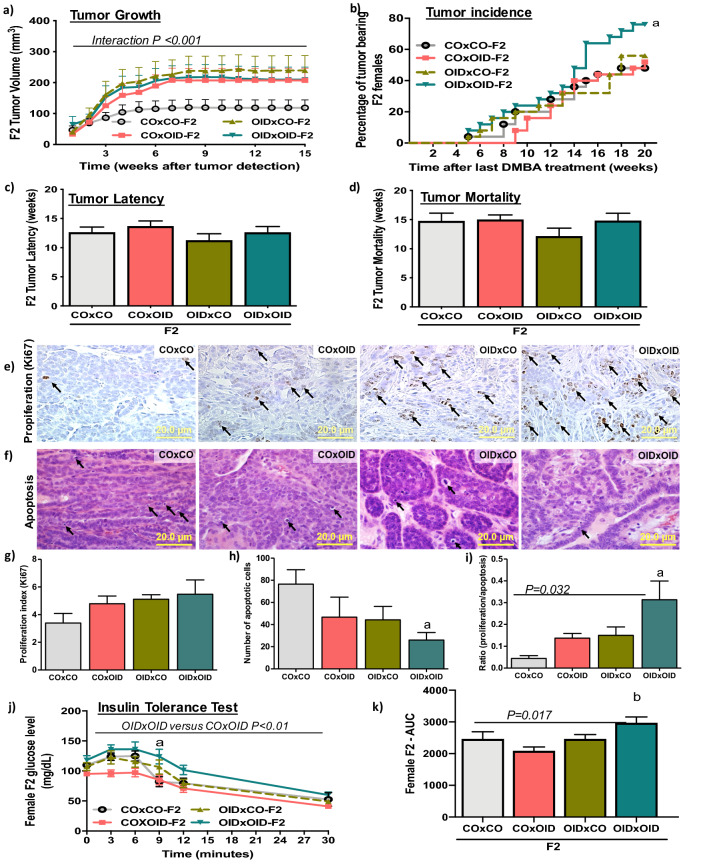
Paternal OID programs breast cancer development and metabolic dysfunction in granddaughters (F2). (**a**–**d**) Carcinogen-induced mammary tumorigenesis in CO and OID female F2 offspring. Mammary tumor growth (volume, mm^3^; **a**), tumor incidence (**b**), tumor latency (**c**) and tumor mortality (**d**) (n = 25/group). Photomicrograph of Ki-67 immunostaining (**e**) (20×, staining indicated by arrows) and apoptotic cells (**f**) (H&E morphological assessment, 20×, cells indicated by arrows). Graphs below show proliferation index (**g**), number of apoptotic cells (**h**), and proliferation/apoptosis ratio (**i**), (**e**–**i**, n = 6/group). Insulin tolerance test (ITT) (**j**) and (**k**) area under curve (AUC) in CO and OID female F2 offspring (n = 8/group). Tumor incidence is shown as percentage of animals with tumors. All other data are mean ± SEM. Significant difference were determined by Kaplan–Meier analysis followed by log-rank test (tumor incidence), repeated measures ANOVA (mammary tumor volume), one-way ANOVA (tumor latency, mortality, proliferation, apoptosis and area under curve), or two-way ANOVA (ITT) followed by post-hoc analysis. “a” indicates statistically significant difference (*P* ≤ 0.05) between OIDxOID and COxCO; “b” indicates statistically significant difference (*P* ≤ 0.01) between OIDxOID and COxOID.

Given the tRF alterations observed in the F1 OID offspring germline and their documented role in the transmission of environmentally induced epigenetic inheritance of disease^3,8^, we then asked whether breast cancer predisposition in OID daughters could be inherited by a second generation of females. To answer this question, we produced the F2 generation by mating F1 male offspring from OID fathers with F1 females from either CO [OIDxCO] or OID [OIDxOID] groups. Similarly, F1 male offspring from CO fathers were mated with F1 females from either the CO [COxCO)] or OID [COxOID] groups. Indeed, we found that the female F2 generation derived from either the F1 OID male and female lineage (OIDxCO and COxOID, respectively) or both (OIDxOID) developed carcinogen-induced mammary tumors that grew significantly faster, compared to COxCO group (*P* < 0.001, Fig. 5a). The incidence of mammary tumors at the end of the monitoring period was also significantly higher in F2 OIDxOID females compared to the COxCO group (*P* = 0.037; Fig. 5b). Tumor latency and tumor mortality rates in the OIDxCO group were slightly shorter than in all other groups, however results did not reach statistical significance (Fig. 5c,d).

Given the increased tumor growth in the F2 generation derived from the OID lineage (Fig. 5a), we next investigated cell proliferation and apoptosis rates in F2 mammary tumors (Fig. 5e–i.) We observed a non-significant increase in cell proliferation and a decrease in apoptotic indices in F2 females tumors derived from the OID lineage compared to COXCO F2 offspring (Fig. 5g,h). Overall, tumors from OID groups (OIDxCO and COxOID, OIDxOID) also showed significantly higher proliferation/apoptosis ratio (*P* = 0.032; Fig. 5i). Between-group comparisons showed that the OIDxOID group had significantly lower tumor apoptosis levels and higher tumor proliferation/apoptosis ratio compared to the COxCO group (*P* = 0.013 and *P* = 0.004, respectively; Fig. 5h,i).

While all F2 females derived from the OID grand-paternal lineage (COxOID, OIDxCO, OIDxOID) showed higher mammary tumor growth with significantly larger tumors (Fig. 5a) when compared to COxCO, only OIDxOID females developed impaired insulin sensitivity as shown by higher glucose levels in ITT and AUC values (*P* = 0.007, *P* = 0.017, Fig. 5j,k). However, OIDxCO females were significantly heavier overtime compared to all other groups (COxCO, COxOID, OIDxOID, *P* = 0.0004, Fig. S3).

## Discussion

We previously reported that paternal obesity increases tumorigenesis in offspring, including breast cancer in rodent models^15,16,18^. Our findings in this follow-up study suggest that metabolic disturbances in the F1 generation play a key role in the enhanced mammary cell growth observed in offspring of obese fathers and help explain why they are more prone to cancer development. We also report that the paternal obesity leads to higher cancer development in two successive generations of offspring. Transmission of the increased breast cancer phenotype into the F2 generation was associated with epigenetic changes in tRFs in the OID male germline.

In previously published reports, we showed that daughters of obese fathers had both metabolic dysfunction and mammary gland abnormalities^15,16^. Thus, our first aim was to dissect the distinct contributions of the systemic effects and local tissue-confined factors on mammary gland and breast cancer development in daughters of obese fathers. Our results suggest that systemic metabolic changes in OID daughters—likely acting though the mammary stroma— play a larger role than alterations in the mammary epithelium itself. We showed that mammary tissues from CO offspring transplanted into OID daughters acquired a growth advantage compared to those transplanted in CO, suggesting that the stroma in OID females allows for better mammary implantation and growth. Similarly, mammary tumors of CO offspring transplanted into OID daughters showed increased rates of cell proliferation/survival, despite the lack of significant differences in tumor growth between groups. In line with that, studies have highlighted the importance the stroma microenvironment on normal mammary development and malignant transformation of the mammary epithelium^23–26^. It is also well established in epidemiologic studies that metabolic conditions such as obesity, metabolic syndrome and diabetes are important risk factors for breast cancer and other malignancies^27–30^ and data from animal models support those findings in humans^31,32^.

Although we did not directly investigated the molecular mechanisms behind the increased cancer development in the progeny of obese fathers in the current study, it is known that metabolic dysfunction contributes to cancer growth via extrinsic and tumor-intrinsic factors^33^. Indeed, we previously reported that paternal obesity or paternal malnutrition alters the molecular make-up of offspring’s mammary tumors which displayed increased signaling of growth factor and energy sensing pathways and altered amino-acid metabolism^15–18^.

The second aim of our study was to examine whether the offspring’s breast cancer predisposition programmed by paternal obesity could be transmitted to a second generation. We found that breast cancer predisposition observed in OID daughters is passed down to the OID granddaughters either via the F1 male and female germlines or both. Our finding are in agreement with a recent study showing that grand-paternal nutritional status is linked to cancer mortality in grandchildren in human populations^21^. OID granddaughters also developed metabolic dysfunction but only when both their progenitors were children of obese fathers.

We also found that both the F0 and F1 male germline had alterations in tRFs, a class of small non-coding RNAs abundant in sperm, recently shown to transmit environmentally-induced information from one generation to another^7,8^. Interestingly, there was an overlap in five tRFs altered in sperm of F1 and F0 males. This suggests either that the F1 male germline is programmed by paternal obesity or that sperm non-coding RNAs are re-set in the F1 generation likely in response to their own metabolic dysfunction. Unfortunately, the inherent scarcity and technical challenges in collecting the female germline prevented us from evaluating its molecular make-up. However, given that both the F1 male and female OID germline were able to transmit the increased predisposition to breast cancer phenotype to a second generation, epigenetic changes in the female germline likely occurred as well.

While details on the functional role of sperm tRFs are still under investigation, these small RNAs have been implicated in different biological processes including regulation of gene expression at the transcriptional, post-transcriptional, and translational level^6,34,35^. Given the short half-life of sperm tRFs, it has been proposed that they act very early in embryonic development post-fertilization, setting a cascade of molecular events which biases cellular programming during subsequent divisions and culminate in disease phenotypes^3,6^. In line with that, our gene ontology analysis of targets of the five overlapping tRFs in OID F0 and F1 males’ sperm showed an enrichment for functions associated with embryonic development including DNA binding, transcription factor activity, transcriptional regulation, and transmembrane transporters. However, whether tRFs altered in OID F0 and F1 males germline play a functional role in breast cancer phenotypes in the F1 and F2 generations remains to be investigated in follow-up studies.

In conclusion, the findings described here build on our previous work and suggest that systemic alterations in offspring of obese fathers play a prominent role in their mammary development and cancer growth. Importantly, the effects of paternal obesity on breast cancer development persist for at least two generations. This evidence for a multigenerational effect on cancer development in our animal model is reinforced by a recent publication showing an association between grandpaternal nutritional status and progeny cancer mortality in human populations^21^. This notion is also supported by our prior findings showing that maternal exposure to an endocrine disruptor or dietary fat can also lead to multigenerational risk of breast cancer through both the male and female germlines in rats^12^. Finally, it is important to note that conditions such as obesity and malnutrition often occur in minorities and disadvantaged populations^36^. Our findings suggest that social determinants of cancer predisposition and outcomes may be imprinted even before birth and can be epigenetically mediated. However, it remains to be determined whether the biological insights uncovered by our study can account for inherited cancer predisposition or cancer disparities in humans.

## Material and methods

### Dietary exposures and breeding

The C57BL/6NTac mouse strain (Taconic BioSciences) was used in all experiments. Male mice were randomly assigned to AIN93G-based diets containing either 17.2% (Control, CO, Envigo-Teklad #TD160018) or 57.1% (Lard-based, Obesity-Inducing-Diet, OID, Envigo-Teklad #TD160019) energy from fat (Diet details in supplementary Table S2, see the section on supplementary data) starting after weaning (3 weeks of age). Males’ body weight was recorded weekly (Fig. S4). At 10 weeks of age, OID-fed and CO-fed F0 male mice were mated with female mice reared solely on the CO diet to generate the F1 generation. Males were kept in female cages for 3 days. Female mice were kept on the CO diet during the breeding period, for the extent of pregnancy (21 days) and after giving birth. The birth weight and number of pups per litter were determined 2 days after birth. To avoid litter-effect, pups were cross-fostered 1 day after dams gave birth. Pups from 2 to 3 dams were pooled and housed in a litter of 8–10 pups per nursing dam. All pups were weaned on postnatal day 21 and fed the CO diet throughout the experiment. Pups body weight was recorded weekly.

To obtain the F2 generation, F1 male offspring from OID fathers were mated with F1 females from either CO [OIDxCO] or OID [OIDxOID] groups. Similarly, F1 male offspring from CO fathers were mated with F1 females from either the CO [COxCO)] or OID [COxOID] groups. No sibling mating was carried out. F1 and F2 generation females from the CO or OID lineages were used to study body weight, metabolic function, mammary tumorigenesis and mammary transplantation, as described in the following sections. The experimental design is shown in Fig. S5.

F1 and F2 litters’ gender distribution and number of offspring used in each experiment are shown in Tables S3 and S4, respectively.

All animal procedures were approved by the Georgetown University Animal Care and Use Committee, and the experiments were performed following the National Institutes of Health guidelines for the proper and humane use of animals in biomedical research. To increase rigor and reproducibility, animals were randomized to each experimental condition/ experiment and studies performed blindly where possible and according to the ARRIVE Essential 10 guidelines.

At the end of the experimental timeline, mice were euthanized by CO_2_ exposure consistent with the recommendations of the Guidelines of Euthanasia (2013) set forth by the American Veterinary Medical Association.

### Metabolic function

Insulin tolerance test (ITT) was performed after the mice fasted for 6 h, according to the method described by Takada et al.^37^. The insulin load (75 mU/100 g body weight) was injected as a bolus, and the blood glucose levels were determined at 0, 3, 6, 9, 12, and 30 min after injection in female offspring. The area under the curve (AUC) was calculated according to the trapezoid rule. Differences in ITT were analyzed using two-way ANOVA (group, time), followed by post-hoc analyses.

### Mammary transplantation

Three-week old F1 female offspring of CO and OID males underwent a mammary gland transplantation surgery as previously described^38,39^. The experimental design is shown in Fig. S6. Females undergoing surgery were anesthetized using isoflurane flowing 3–5% in oxygen (0.5 L/min), and maintained with isoflurane flowing at 1–3%. At the end of the procedure, before discontinuing the inhalation-anesthesia, the animal was injected s.c. with the analgesic buprenorphine (0.5 mg/kg). A second dose of buprenorphine was injected 6–8 h after the first dose. Animals were checked daily thereafter for a week.

Before transplantation, the 4th inguinal mammary gland of host females was cleared from their endogenous epithelium by removing the fat pad of the 4th gland up to its proximal lymph node. Special care was taken to cut off the connection between the 4th and 5th mammary glands to ensure complete clearing of the 4th mammary fat pad and to avoid later epithelial contamination from the 5th mammary gland. The excised fat pad containing the epithelial cells were stained with carmine aluminum solution to check cleared margins.

For transplantation, the donor fat pad containing the epithelial cells was excised and divided into small pieces (1 mm^3^) and placed into a tissue-culture plate containing DMEM/F12 to keep it moist. Mammary tissue fragments of the donor mouse, either CO or OID F1 female offspring, were then implanted into a pocket made in the cleared fat pad of the host (CO or OID). The skin incision was closed with surgical wound clips. The transplantations were performed from CO female offspring donors to both CO [CO(CO-MG)] and OID [OID(CO-MG)] female offspring hosts, as well as from OID female offspring donors to CO [CO(OID-MG)] female offspring hosts. Mammary glands transplants were collected approximately 10 weeks post-surgery and used for analysis of epithelial branching density, epithelial elongation and number of Terminal End Buds (TEBs) as described in the next sections.

### Transplanted mammary gland growth and development

Transplanted mammary glands collected approximately 10 weeks post-surgery were stretched onto a slide, placed in a fixative solution and stained with a carmine aluminum solution (Sigma Chemical Co.) as previously described^40^. Whole mounts were examined under the microscope (AmScope) for ductal elongation and number of TEBs (undifferentiated structure considered to be the targets of malignant transformation), as previously described^40^. Whole-mount slides were also photographed (Olympus SZX12 250 Stereomicroscope), digitized and analyzed. Briefly, the portion surrounding the glandular epithelium was removed, color channels separated, and noise removed. The images were thresholded and skeletonized. Then, mammary epithelial area and branching (sum of intersections) were measured by Sholl analysis, a plugin ImageJ software (National Institute of Health, Bethesda, MD, USA) as previously described^41^. Once morphological analyses were completed, mammary whole mounts were removed from the slide, embedded in paraffin, sectioned (5 µm)^42^ and prepared for either hematoxylin and eosin (H&E) or ki-67 staining as described below. Differences between groups were analyzed using one-way ANOVA, followed by post-hoc analyses.

### Mammary tumor induction

Mammary tumors were induced in F1 an F2 female offspring by administration of medroxyprogesterone acetate (MPA; 15 mg/100 µl, subcutaneously) to 6 weeks of age female offspring, followed by 3 weekly doses of 1 mg 7,12-dimethylbenz[a]anthracene (DMBA; Sigma, St. Louis, MO) dissolved in peanut oil by oral gavage^43^. Tumors were detected by palpation once per week, starting at week 2 after the last dose of DMBA. Tumor growth was measured using a caliper, and the width and height of each tumor were recorded.

In the F1 generation, mammary tumors were harvested when reaching approximately 40 mm^2^ in volume and used for mammary tumor transplantation surgery, as described in the next section. In the F2 generation, tumor development was monitored for a total of 20 weeks post-DMBA administrations. Animals in which tumor burden reached approximated 10% of total body weight were euthanized before the end of the monitoring period, as required by our institution. Tumor growth was analyzed using two-way ANOVA (group and time), followed by post-hoc analyses. Kaplan–Meier survival curves were used to compare differences in tumor incidence, followed by the log-rank test. Differences in tumor latency and mortality were analyzed using two-way ANOVA.

All DMBA-induced tumors in F1 and F2 females were blindingly evaluated by a consultant pathologist, Dr. Susana Galli. Only mammary carcinomas were included in the final analysis or used in mammary tumor transplantation below.

### Mammary tumor transplantation

CO and OID F1 female offspring underwent a mammary tumor transplantation surgery at approximately 11 weeks of age. Females undergoing this procedure were anesthetized and treated with and analgesic post-surgery as described under the *Mammary Transplantation* section. Briefly, carcinogen-induced mammary tumor fragments (1 mm^3^) of a donor mouse, either CO or OID offspring, were implanted into a pocket made in the mammary fat pad of the host (CO or OID). The experimental design is shown in Fig. S6. Mammary tumors grown from the transplants were collected approximately 6–8 weeks post-surgery. Differences between groups were analyzed using one-way ANOVA, followed by post-hoc analyses.

### Analysis of cell proliferation

Cell proliferation (Ki-67) was evaluated by immunohistochemistry in F1 mammary gland and mammary tumors transplants as well as F2 mammary tumors. Briefly, tissues were fixed in 10% buffered formalin, embedded in paraffin, and sectioned (5 µm). Sections were deparaffinized with xylene and rehydrated through a graded alcohol series. Antigen retrieval was performed by immersing the tissue sections at 98 °C for 40 min in 1× Diva Decloaker (Biocare). Tissue sections were treated with 3% hydrogen peroxide and 10% normal goat serum for 10 min and were incubated with the primary antibody, overnight at 4 °C. After several washes, sections were treated to the appropriate HRP labeled polymer for 30 min and DAB chromagen (Dako) for 5 min. Slides were counterstained with Hematoxylin (Fisher, Harris Modified Hematoxylin), blued in 1% ammonium hydroxide, dehydrated, and mounted with Acrymount. The sections were photographed using an Olympus IX-71 Inverted Epifluorescence microscope at 40× magnification. Proliferation index (Ki-67 staining) was determined by immunoRatio, a plugin Image J software (National Institute of Health, Bethesda, MD, USA), to quantify hematoxylin and DAB-stained cells. Differences between groups were analyzed using one-way ANOVA, followed by post-hoc analyses.

### Analysis of cell apoptosis

Cell apoptosis analysis was performed in F1 transplanted mammary glands and tumors and F2 mammary tumors by morphological detection. Tissues were fixed in neutral buffered 10% formalin, embedded in paraffin, sectioned (5 µm) and stained with hematoxylin and eosin (H&E). Cells presenting loss of adhesion between adjacent cells, cytoplasmic condensation and formation of apoptotic bodies were considered apoptotic as described before^44^. Sections were photographed using an Olympus IX-71 Epifluorescence microscope at 40× magnification. Twenty areas were photographed randomly, and the number of apoptotic bodies counted. Images were evaluated with ImageJ software (NIH, USA). Differences between groups were analyzed using one-way ANOVA, followed by post-hoc analyses.

### Mature spermatozoa collection and purification

CO and OID-fed males (F0) and their male offspring (F1) were euthanized and their caudal epididymis dissected for sperm collection. The epididymis was collected, punctured, and transferred to tissue culture dish containing M2 media (M2 Medium-with HEPES, without penicillin and streptomycin, liquid, sterile-filtered, suitable for mouse embryo, Sigma, product #M7167) where it was incubated for 1 h at 37 °C. Sperm samples were isolated and purified from somatic cells. Briefly, the samples were washed with PBS, and then incubated with SCLB (somatic cell lysis buffer, 0.1% SDS, 0.5% TX-100 in Diethylpyrocarbonate water) for 1 h. SCLB was rinsed off with 2 washes of PBS and the somatic cell-free purified spermatozoa sample pelleted and used for RNA extraction.

### Small RNA-seq and gene ontology (GO) analyses

Total RNA was isolated from sperm using Qiagen’s miRNeasy extraction kit, according to the manufacturer’s instructions. One hundred ng of column-purified sperm RNA was used to prepare individually barcoded small-RNA libraries. Samples were barcoded, pooled, precipitated and separated on a 15% polyacrylamide gel (PAGE). The gel was stained with SYBR gold dye and the small non-coding RNA segment corresponding to transfer RNA fragments or tRFs (30–45 nucleotides) excised and purified using a cDNA library preparation method described previously^45^. This library preparation method was demonstrated to be highly reproducible using total RNA with RNA Integrity Numbers as low as 2.0^45^. Indexed, single-ended small-RNA sequencing libraries were prepared. For each individual barcoded library, at least 10 million reads (raw data) were generated using an Illumina Hi-Seq 2500. The raw reads were subjected to 3′ adapter trimming and low quality filtering using Trimmomatic program^46^. The high quality clean reads (Data quality control is shown in Fig. S7) were aligned to the mouse genome. tRFs tags were mapped to the mouse genome (GRCm38/mm10 reference genome) in order to analyze their genomic distribution and expression in the different sperm RNA samples. Two different types of reads that were generated during the alignment process: unique reads and shared reads. We calculated weighted reads based on these two reads by using the following formula "weighted reads = unique reads + alignment score % * shared reads". (Alignment score quantifies the similarity between two sequences). The weighted reads were used for all downstream analysis as raw reads for each tRNA. Small RNA tags were annotated and aligned to known t-RNA sequences using Ref-seq, GenBank and Rfam database using blastn with standard parameters. To analyze the differential expression of tRFs between CO and OID groups, tRFs were normalized to TPM (Transcripts Per Kilobase Million). tRFs with a P value less than 0.05 were considered significant, with an appropriate correction for multiple testing^47^. Target genes for the 5 overlapping tRFs in OID F0 and F1 males were predicted using TargetScan Mouse custom seedmatch and modified miRanda algorithm (energy ≤ − 20 and score ≥ 150). The common predicted genes were then uploaded to PANTHER 15.0 for GO term and pathway analysis, final lists were filtered by FDR < 0.25.

### Ethics approval

All animal procedures were approved by the Georgetown University Animal Care and Use Committee (protocol # 2016-1172), and the experiments were performed following the National Institutes of Health guidelines for the proper and humane use of animals in biomedical research.

## Supplementary Information

Supplementary Information

## Data Availability

The small RNA-seq data has been deposited in GEO (Gene Expression Omnibus) database with accession code GSE161831.

## Abbreviations

AUC: Area under the curve
BRCA: Breast Cancer gene
CO: Control diet
DMBA: 7,12 Dimethylbenz[a]anthracene
F0: Parental generation
F1: First filial generation
F2: Second filial generation
GO: Gene ontology
H&E: Hematoxylin and eosin
ITT: Insulin tolerance test
MPA: Medroxyprogesterone acetate
OID: Obesity-inducing diet
SCLB: Somatic cell lysis buffer
TEBs: Terminal end buds
tRFs: tRNA-derived fragments
OID daughters/females: Daughters of OID fed males (daughers were kept in control chow throughout the study)
CO daughters/females: Daughters of CO diet fed males
[CO(CO-MG)]: Mammary gland from F1 CO female offspring transplanted into CO female offspring
[CO(OID-MG)]: Mammary gland from F1 OID female offspring transplanted into CO female offspring
[OID(CO-MG)]: Mammary tumor from F1 CO female offspring transplanted into OID female offspring
[CO(OID.T)]: Mammary tumor from F1 OID female offspring transplanted into CO female offspring
[CO(CO.T)]: Mammary tumor from F1 CO female offspring transplanted into CO female offspring.
[OID(CO.T)]: Mammary tumor from F1 CO female offspring transplanted into OID female offspring.
COxCO: Granddaughters of CO diet fed males
OIDxCO: Granddaughters of OID fed males through the male germline
COxOID: Granddaughters of OID fed males through the female germline
OIDxOID: Granddaughters of OID fed males through both the male and female germlines
