# Supplementary material for: Systemic alterations play a dominant role in epigenetic predisposition to breast cancer in offspring of obese fathers and is transmitted to a second generation
Source: Sci Rep. 2021 Apr 1;11:7317. doi: 10.1038/s41598-021-86548-w (PMC8016877; doi:10.1038/s41598-021-86548-w)
Supplement: Supplementary file 1 — Supplementary Information [file 41598_2021_86548_MOESM1_ESM.docx]

**SUPPLEMENTARY INFORMATION FOR:**

**Systemic alterations play a dominant role in epigenetic predisposition to breast cancer in offspring of obese fathers and is transmitted to a second generation**

**Camile C. Fontelles^1^, Raquel Santana da Cruz^1^, Alexandra K. Gonsiewski^1^, Ersilia Barin^1^, Volkan Tekmen^1^, Lu Jin^1^, M. Idalia Cruz^1^, Olivier Loudig^1,2^, Anni Warri^3^* and Sonia de Assis^1^***

^1^Department of Oncology, Lombardi Comprehensive Cancer Center, Georgetown University, Washington, DC, United States.

^2^ Center for Discovery and Innovation, Hackensack Meridian Health (HMH), Nutley, NJ, United States.

^3^ Institute of Biomedicine, University of Turku Medical Faculty, Turku, Finland.

***Corresponding authors:**

Sonia de Assis, Ph.D., 3970 Reservoir Road, NW, The Research Building, Room E410, Washington, DC 20057, USA; Phone: (202) 687-9518, Fax: (202) 687-7505, Email: [deassiss@georgetown.edu](mailto:deassiss@georgetown.edu), ORCID: 0000-0001-5053-0614

Anni Warri, PhD., Institute of Biomedicine, University of Turku Medical Faculty, Turku, 20014 Finland, Email: awarri@utu.fi

**Supplementary Figures Legends**

**Figure S1: OID and CO F1 generation offspring’s body weight at different stages of life**. Birth (**a**), weaning (**b**), and longitudinal body weight in female (n=25/group) (**c**) and male (n=34-43/group) (d) F1 generation offspring from fathers fed with CO and OID diets. The data are expressed as mean ± SEM. Significant differences versus the control group were determined by two-way followed by post-hoc analysis. *P≤0.05; **P≤0.01; ***P≤0.001.

**Figure S2: Paternal OID reprograms the sperm small non-coding RNA load in fathers (F0) and sons (F1).** Levels (percentage reads) of the 5 tRFs with overlapping significant differential expression in both OID fathers(F0) and sons (F1) compared to CO (n=3-4/group).

**Figure S3:** **OID and CO F2 generation offspring’s body weight at different stages of life**. Birth (**a**)and weaning (**b**) of male and female offspring; Longitudinal body weight (**c**) of female F2 offspring (n=25/group). The data are expressed as mean ± SEM. Significant differences were determined by two-way ANOVA followed by post-hoc analysis. “a” indicates statistically significant difference (P≤0.05) between OIDxCO and COxCO group; “b” indicates statistically significant difference (P≤0.05) between OIDxCO and OIDxOID; “c” indicates statistically significant difference (P≤0.05) between OIDxCO and COxCO, OIDxCO and OIDxOID.

**Figure S4:** **Paternal (male F0) body weight gain:** Longitudinal body weight in control (CO, n=12) and obesity-inducing diet (OID, n=11) fed male mice sires (F0). The data are expressed as mean ± SEM. Significant differences versus the control group were determined by two-way ANOVA followed by post-hoc analysis. *P≤0.05; **P≤0.01; ***P≤0.001

**Figure S5:** **Breeding scheme to produce the F1 and F2 generations:** Male mice were fed the experimental diets [control (CO) or obesity-inducing diet (OID)] from 3 to 10 weeks of age. CO diet or an OID diet-fed male mice (**F0**) were mated with female mice that were reared on a CO diet only. The resulting male and female offspring (**F1**) were used to produce the **F2** generation. No sibling mating was carried out.

**Figure S6:** **Mammary transplantation study design:** **a**) CO diet or an OID diet-fed male mice (F0) were mated with female mice (F0) that were reared on a CO diet only. F1 females, which consumed only CO diet, were submitted to either a **Mammary Transplantation** (M.T.) or to a **Tumor Transplantation** (T.T.). **b**) For the M.T., female recipients (from both CO and OID groups) had their 4th inguinal mammary gland removed (1) and later received a mammary gland transplant (colored circles) (2) from either a donor from the same group or from the opposite group. **c**) For the T.T., female donors received 7,12-dimethylbenz[a]anthracene (DMBA) to induce mammary tumors. Later, female recipients (from both CO and OID groups) received, in their 4th inguinal mammary gland, a tumor transplant (colored triangles) from either a donor from the opposite or from the same group.

**Figure S7: Small RNA-seq data quality control measures.** Per base sequence quality whisker plot: distribution of quality of bases for raw RNA-seq data using FastQC. Each plot represents a CO or OID sperm sample from the F0 (**a**, fathers) or F1 (**b**, sons) generations.  Lower quality reads were trimmed before analysis.

**Supplementary Figures and Tables**

**Figure S1**

**Figure S2**

**Figure S3**

**Figure S4**

**Figure S5**

**Figure S6**

**a)**

**b) Figure S7**

**Table S1:** Mammary gland development in 3-week old female offspring of CO and OID male mice.

| **Parameters** | **CO** | **OID** |
| --- | --- | --- |
| **Epithelial Branching** | 2.0±0.3 | 2.6±0.4 |
| **Number of TEBs** | 5.4±1.0 | 6.0±1.0 |
| **Epithelial Elongation (cm)** | 0.30±0.03 | **0.41±0.03*** |

All data are mean ± SEM (n=6/group). *P≤0.05 by t-test.

**Table S2:** Composition of the experimental diets

| *Ingredients* | *g/kg* | |
| --- | --- | --- |
|  | **Control (CO)**  **TD.160018** | **Obesity-inducing diet (OID)**  **TD.160019** |
| Casein | 200.0 | 288.0 |
| L-Cystine | 3.0 | 2.0 |
| Corn Starch | 397.386 | - |
| Maltodextrin | 132.0 | 150.45 |
| Sucrose | 100.0 | 142.0 |
| Corn Oil | 50.0 | 50.0 |
| Lard | 20.0 | 280.0 |
| Cellulose | 50.0 | 20.0 |
| Mineral Mix, AIN-93-MX (94046) | 35.0 | 49.7 |
| Vitamin Mix, AIN-93-VX (94047) | 10.0 | 14.2 |
| Choline Bitartrate | 2.50 | 3.55 |
| TBHQ, antioxidant | 0.014 | - |
| Food Coloring | 0.1 | 0.1 |
| % protein by weight **(% kcal**) | 17.7 (**18.8**) | 25.3 (**19.3**) |
| % carbohydrate by weight (**% kcal**) | 60.1 (**63.9**) | 31.0 (**23.6**) |
| % fat by weight (**% kcal**) | 7.2 (**17.2**) | 33.3 (**57.1**) |
| **Kcal/g** | **3.8** | **5.2** |

**Table S3:** Proportion of female and male offspring in CO and OID litters.

| **Generation** | **Group** | **Female** | **Male** |
| --- | --- | --- | --- |
| **F1** | **CO** | 0.57±0.2 | 0.43±0.1 |
|  | **OID** | 0.49±0.2 | 0.51±0.2 |
| **F2** | **COxCO** | 0.57±0.5 | 0.43±0.4 |
|  | **COxOID** | 0.45±0.6 | 0.55±0.4 |
|  | **OIDxCO** | 0.52±0.4 | 0.48±0.4 |
|  | **OIDxOID** | 0.56±0.6 | 0.44±0.5 |

All data are mean ± SEM.

**Table S4:** Number of offspring and number of contributing fathers per experiment.

| **Samples** | **Assay** | **Group** | **Father** | **Female** |
| --- | --- | --- | --- | --- |
| **F1 offspring** | **Insulin Tolerance Test** | **CO** | 5 | 6 |
|  |  | **OID** | 4 | 6 |
| **Mammary gland transplantation of female F1 offspring** | **Mammary gland development** | **CO(CO)** | 5 | 5 |
|  |  | **CO(OID)** | 6 | 9 |
|  |  | **OID(CO)** | 10 | 12 |
| **Mammary tumor transplantation of female F1 offspring** | **Tumorigenesis** | **CO(CO)** | 7 | 10 |
|  |  | **CO(OID)** | 7 | 12 |
|  |  | **OID(CO)** | 6 | 18 |
|  | **Ki67** | **CO(CO)** | 5 | 5 |
|  |  | **CO(OID)** | 4 | 5 |
|  |  | **OID(CO)** | 4 | 9 |
|  | **Apoptosis** | **CO(CO)** | 4 | 5 |
|  |  | **CO(OID)** | 3 | 3 |
|  |  | **OID(CO)** | 6 | 11 |
| **F2 offspring** | **Ki67** | **COxCO** | 5 | 6 |
|  |  | **COxOID** | 6 | 6 |
|  |  | **OIDxCO** | 4 | 6 |
|  |  | **OIDxOID** | 4 | 6 |
|  | **Apoptosis** | **COxCO** | 5 | 6 |
|  |  | **COxOID** | 6 | 6 |
|  |  | **OIDxCO** | 4 | 6 |
|  |  | **OIDxOID** | 4 | 6 |
|  | **Insulin Tolerance Test** | **COxCO** | 5 | 8 |
|  |  | **COxOID** | 6 | 8 |
|  |  | **OIDxCO** | 4 | 8 |
|  |  | **OIDxOID** | 4 | 8 |
|  | **Tumorigenesis** | **COxCO** | 10 | 25 |
|  |  | **COxOID** | 10 | 25 |
|  |  | **OIDxCO** | 10 | 25 |
|  |  | **OIDxOID** | 7 | 25 |
